# The posterior *HOXD* locus: Its contribution to phenotype and malignancy of Ewing sarcoma

**DOI:** 10.18632/oncotarget.9702

**Published:** 2016-05-30

**Authors:** Kristina von Heyking, Laura Roth, Miriam Ertl, Oxana Schmidt, Julia Calzada-Wack, Frauke Neff, Elizabeth R. Lawlor, Stefan Burdach, Günther H.S. Richter

**Affiliations:** ^1^ Laboratory for Functional Genomics and Transplantation Biology, Children's Cancer Research Center and Department of Pediatrics, Klinikum rechts der Isar, Technische Universität München, Munich Comprehensive Cancer Center (CCCM), and German Translational Cancer Research Consortium (DKTK), Munich, Germany; ^2^ Institute of Pathology, Helmholtz Zentrum München - German Research Center for Environmental Health (GmbH), Neuherberg, Germany; ^3^ Departments of Pediatrics and Pathology, University of Michigan, Ann Arbor, Michigan, United States of America

**Keywords:** HOXD, metastasis, Ewing sarcoma, WNT signaling, endochondral development

## Abstract

Microarray analysis revealed genes of the posterior HOXD locus normally involved in bone formation to be over-expressed in primary Ewing sarcoma (ES). The expression of posterior *HOXD* genes was not influenced via ES pathognomonic EWS/ETS translocations. However, knock down of the dickkopf WNT signaling pathway inhibitor 2 (DKK2) resulted in a significant suppression of HOXD10, HOXD11 and HOXD13 while over-expression of DKK2 and stimulation with factors of the WNT signaling pathway such as WNT3a, WNT5a or WNT11 increased their expression. RNA interference demonstrated that individual *HOXD* genes promoted chondrogenic differentiation potential, and enhanced expression of the bone-associated gene *RUNX2*. Furthermore, *HOXD* genes increased the level of the osteoblast- and osteoclast-specific genes, osteocalcin (*BGLAP*) and platelet-derived growth factor beta polypeptide (*PDGFB*), and may further regulate endochondral bone development via induction of parathyroid hormone-like hormone (*PTHLH*). Additionally, HOXD11 and HOXD13 promoted contact independent growth of ES, while *in vitro* invasiveness of ES lines was enhanced by all 3 *HOXD* genes investigated and seemed mediated via matrix metallopeptidase 1 (MMP1). Consequently, knock down of HOXD11 or HOXD13 significantly suppressed lung metastasis in a xeno-transplant model in immune deficient mice, providing overall evidence that posterior *HOXD* genes promote clonogenicity and metastatic potential of ES.

## INTRODUCTION

Ewing sarcomas (ES) are bone or soft tissue tumors with a prominent *stemness* phenotype, mostly occurring in children and adolescents. These highly malignant sarcomas frequently arise in diaphysal bones possibly descending from a neuroectodermal or mesenchymal stem cell in transition from an undifferentiated state to a more differentiated phenotype of the chondro-osseous lineage [1–6]. Genetically, ES are defined by *EWS/ETS* translocations [1, 7, 8].

In the clinical setting, prognosis for patients with metastatic ES at diagnosis is clearly worse than for those without metastases [9]. Especially the development of metastases in bones is a catastrophic event in the clinical course of ES patients [10, 11].

Recently, we demonstrated the pro-metastatic gene *dickkopf WNT signaling pathway inhibitor 2* (*DKK2*) to be critical for malignant growth of ES [5]. It can act as either an agonist or antagonist of WNT/β-catenin signaling, depending on the cellular context and the presence of the co-factor Kremen 2 [12–14]. In ES Kremen 2 is absent and DKK2 stimulates canonical β-catenin signaling. Further, DKK2 promotes bone infiltration and osteolysis *in vivo* and subsequent analyses defined DKK2 as a key factor in osteotropic malignancy [5].

Our subsequent analysis revealed several genes of the *HOXD* cluster to be over-expressed in ES. Class I homeobox genes (*HOX*) are transcription factors known to be involved in embryonic development and body segmentation [15]. In mammals they are organized in 4 different chromosomal loci (HOXA at 7p15.3, HOXB at 17q21.3, HOXC at 12q13.3 and HOXD at 2q31) comprising 39 genes that can be aligned with each other into 13 antero-posterior paralogous groups [16]. *HOX* genes are also expressed in adult human organs [17] where they appear to regulate cell identity [18], cell differentiation [19, 20], including metabolic processes [21]. In addition, posterior *HOXD* genes such as *HOXD11*, *HOXD12* and *HOXD13* were shown to not only regulate patterning but also to directly influence bone formation and the ossification pattern of bones. In part this effect is mediated via runt-related transcription factor 2 (RUNX2) [22]. *HOX* genes are further implicated in neoplastic transformation resulting in leukemia [23] as well as solid cancers derived from various organs [24, 25].

Here we demonstrate posterior *HOXD* genes such as *HOXD10*, *HOXD11* and *HOXD13* are significantly up-regulated in ES. We show that inhibition of DKK2 expression significantly suppresses the expression of HOXD10, HOXD11 and HOXD13 while over-expression of DKK2 and factors stimulating the WNT signaling pathway such as WNT3a, WNT5a or WNT11 further increased their expression. RNA interference of genes of the posterior *HOXD* locus revealed individual *HOXD* genes to be important for chondrogenic differentiation potential. In contrast, osteogenic differentiation was not impacted by *HOXD* gene loss of function but the expression of bone-associated genes such as *RUNX2* was enhanced. Finally, HOXD11 and HOXD13 further promote metastatic growth and invasiveness that seemed to be mediated via matrix metallopeptidase 1 (MMP1).

## RESULTS

### Posterior *HOXD* genes are over-expressed in Ewing sarcoma (ES)

**Figure 1:**
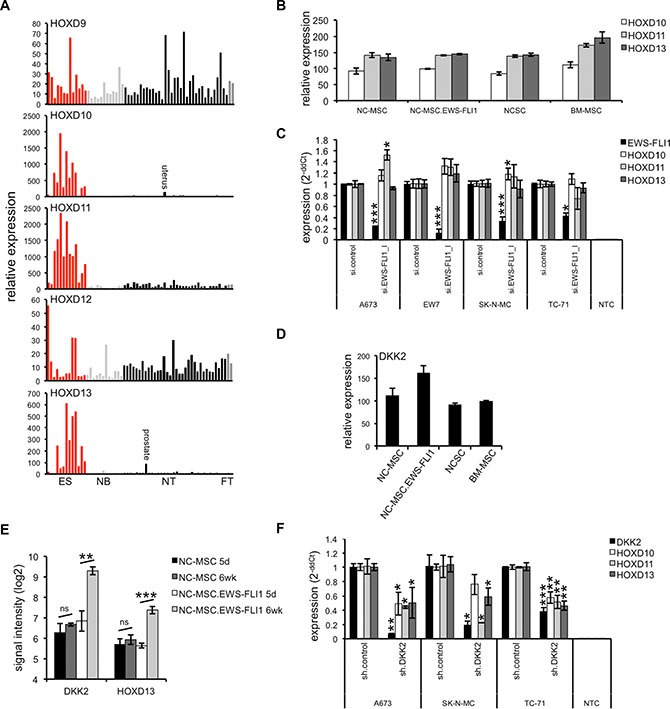
*HOXD* gene expression and regulation in ES (**A**) Expression profile of HOXD9 – HOXD13 in ES (red) in comparison to neuroblastoma (NB; light gray), normal (NT; black) and fetal tissue (FT; dark gray). ES and NB RNA were hybridized onto HG U133A arrays (Affymetrix; GSE1825, GSE15757; [50]) and compared to a published microarray study of normal tissue (GSE2361). Each bar represents the expression signal of an individual array. (**B**) *HOXD* genes seem not induced after over-expression of EWS-FLI1 in mesenchymal and neural crest stem cells. NC-MSC: control vector transduced neural crest-derived MSC after 5 days in self-renewal media, NC-MSC.EWS-FLI1: EWS-FLI1 transduced NC-MSC after 5 days in self-renewal media, NCSC: undifferentiated, freshly isolated neural crest stem cells, BM-MSC: undifferentiated adult bone marrow derived MSC. (**C**) Expression of *HOXD* genes is not affected after suppression of EWS-FLI1 in four different ES lines using RNA interference as measured by qRT-PCR. Data are mean ± SEM; *t*-test. (**D**) Expression of DKK2 in undifferentiated stem cell populations (NC-MSC, NC-MSC.EWS-FLI1, NCSC and BM-MSC; from GEO dataset GEO21511, CEL files were RMA normalized by use of expression console software, Affymetrix). (**E**) Expression of DKK2 and HOXD13 in NC-MSC cells following exposure to differentiation conditions. NC-MSC transduced with GFP-only (NC-MSC) or EWS-FLI1-GFP (NC-MSC.EWS-FLI1) lentiviral vectors were passaged for 5 days in self-renewal media (5 d) and then transferred to differentiation media for 6 weeks (6 wk). Gene expression profiling studies of triplicate samples reveals that exposure to differentiation conditions resulted in up-regulation of DKK2 and HOXD13 in EWS-FLI1^+^ cells. (**F**) Suppression of DKK2 by specific shRNA [5] in different ES lines results in a significantly down-regulation of *HOXD10*, *HOXD11* and *HOXD13* as measured by qRT-PCR. Data are mean ± SEM; *t*-test.

Microarray analysis disclosed genes of the posterior *HOXD* locus to be clearly over-expressed in primary ES. Interestingly, only *HOXD10*, *HOXD11* and *HOXD13* of the homeobox loci, normally involved in bone formation and ossification pattern of bones [22, 26], were significantly up-regulated in ES in comparison to neuroblastoma, normal and fetal tissue (Figure 1A). They were within the 50 most up-regulated genes with the strongest over-expression in ES compared to normal tissue (see Supplementary Material, Table S1). Extended analysis of other sarcoma and carcinoma on publically available expression data revealed that only fibrosarcoma demonstrated a similar up-regulation of genes of the posterior *HOXD* locus (see Supplementary Material, Figure S1A, S1B). Furthermore, qRT-PCR confirmed a significantly lower expression of *HOXD10*, *HOXD11* and *HOXD13* in neuroblastoma and osteosarcoma (see Supplementary Material, Figure S2A).

To investigate the putative origin of this increased *HOXD* gene expression in ES we analyzed public array data of neuroectodermal or mesenchymal stem cells [27] presumed originating for ES. First we recognized a low level of expression of HOXD10, HOXD11 and HOXD13 in neural crest-derived mesenchymal stem cells (NC-MSC) as well as in undifferentiated, freshly isolated neural crest stem cells (NCSC) or adult bone marrow derived MSC (BM-MSC). Interestingly, *HOXD* genes seem not to be further increased after transduction of NC-MSC with EWS-FLI1 (Figure 1B). Neither over-expression of EWS-FLI1 in MSC lines (see Supplementary Material, Figure S2B) nor its knock down in ES lines by specific siRNA (Figure 1C, see Supplementary Material, Figure S2C) [28] did significantly influence posterior *HOXD* gene expression. Furthermore, enhancer of zeste 2 polycomb repressive complex 2 subunit (EZH2) containing PRC2 complex, often involved regulating broad regions of the *HOX* locus during development and in adult tissue [29, 30], does not influence the expression of HOXD10, HOXD11 or HOXD13 in ES (see Supplementary Material, Figure S2D). These observations complement previous results demonstrating absence of H3K27me3 across the *HOXD* cluster in ES [31].

### *HOXD* genes are stimulated via DKK2

Increasing evidence indicates that developmental canonical WNT/β-catenin signaling and *HOX* gene expression may interact: e.g. there are results demonstrating WNT-dependent regulation of HOXB8 in zebrafish during lateral line cell migration [32] or a HOXD13 dependent expression of WNT5a for the regulation of cell polarity in the cartilage growth plate [33]. Recently, we demonstrated DKK2 to be an agonist of the WNT/β-catenin pathway in ES [5]. Here we analyzed whether DKK2 may impact on the expression of posterior *HOXD* genes in ES.

**Figure 2:**
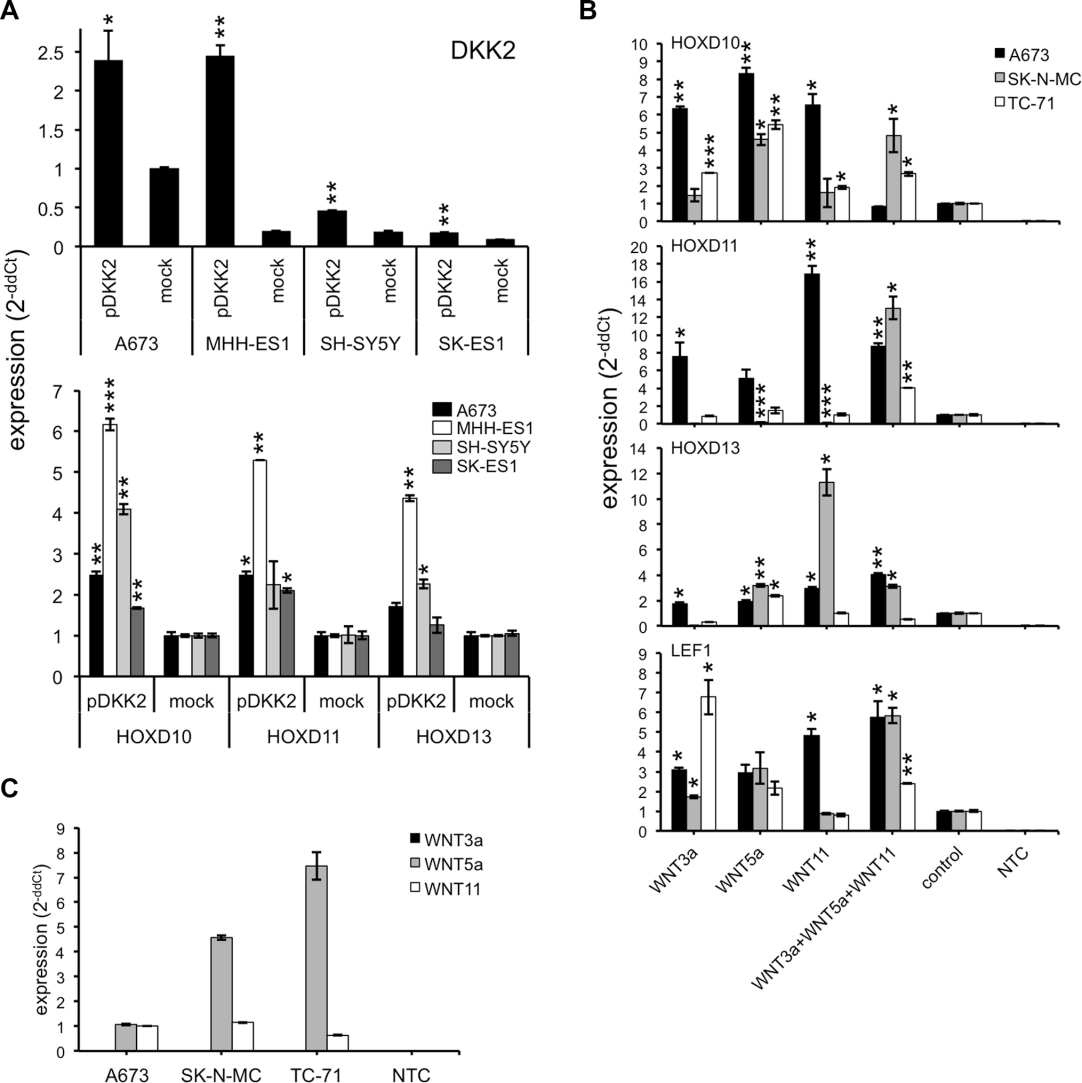
DKK2 increases *HOXD* gene expression through WNT signaling pathway (**A**) Increased *HOXD10*, *HOXD11* or *HOXD13* expression (bottom) after transfection with cDNA encoding human DKK2 (top) in ES lines A673, MHH-ES1, SK-ES1 or NB line SH-SY5Y, respectively. Results of qRT-PCRs are shown. Data are mean ± SEM; *t*-test. (**B**) Analysis of *HOXD* gene expression in A673, SK-N-MC and TC-71 cells after incubation with recombinant human WNT3a, WNT5a or WNT11 and the combination of all three ligands. RNA was isolated after 1, 3, 6 and 12 h and the time point with the highest increase was shown. Data are mean ± SEM; *t*-test. (**C**) Expression of endogenous WNT3a, WNT5a and WNT11 mRNA in three ES cell lines (A673, SK-N-MC and TC-71) analyzed by qRT-PCR. Data are mean ± SEM; *t*-test.

Surprisingly, we first observed an increased DKK2 expression after acute up-regulation of EWS-FLI1 in NC-MSC (Figure 1D). Subsequent expression profiling studies of control and EWS-FLI1^+^ NC-MSC demonstrated that exposure to differentiation conditions for 6 weeks resulted in an EWS-FLI1 dependent up-regulation of DKK2 and HOXD13 in differentiated NC-MSC (Figure 1E, see Supplementary Material, Figure S2E). Consistent with these results, suppression of DKK2 by specific shRNA [5] in different ES lines resulted in a significant down-regulation of HOXD10, HOXD11 and HOXD13 (Figure 1F). Further, down-regulation of TCF4, a transcription factor in the canonical WNT/β-catenin pathway, resulted in a partial inhibition of posterior *HOXD* gene expression (see Supplementary Material, Figure S3A). Thus, the WNT signaling pathway seems to be generally active in ES: (i) over-expression of DKK2 in ES cell lines as well as in the NB cell line SH-SY5Y (Figure 2A) increased posterior *HOXD* gene expression; (ii) treatment of different ES lines with WNT3a, WNT5a or WNT11 or combined resulted in a further induction of *HOXD10*, *HOXD11* or *HOXD13* as well as of the WNT/β-catenin target gene *LEF1* (Figure 2B). This induction was independent of the level of endogenous WNT expression in ES cell lines (Figure 2C and [14]).

### Posterior *HOXD* genes contribute to chondrogenic as well as bone associated gene expression in ES

ES are bone or soft tissue neoplasms with a prominent immature *stemness* phenotype maintained by epigenetic repressors BMI1 and EZH2 [27, 28]. Further, posterior *HOXD* genes regulated by EZH2 during development are known to influence the ossification pattern of bones [22], so it seemed relevant to investigate whether the expression of posterior *HOXD* genes in ES may influence their differentiation capacity.

**Figure 3:**
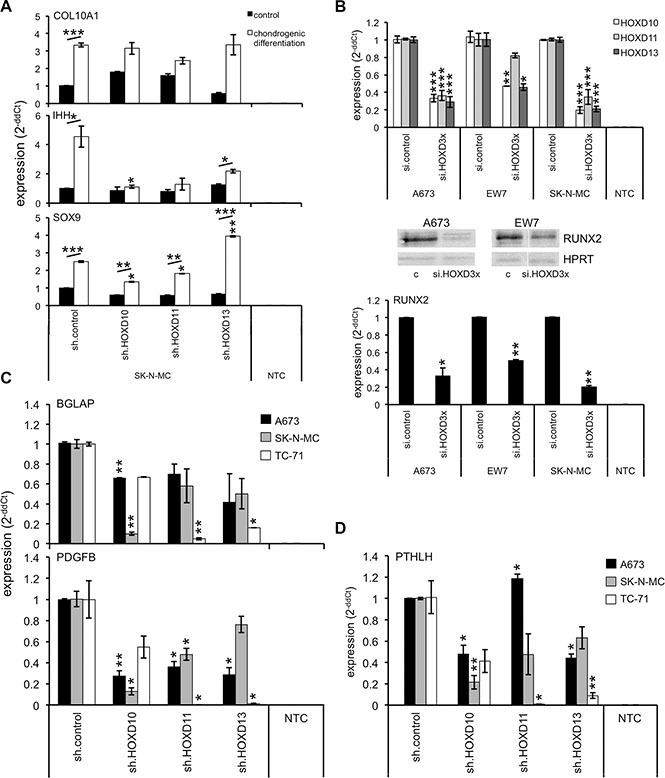
Posterior *HOXD* genes promote chondrogenic differentiation and expression of bone associated genes (**A**) Chondrogenic differentiation potential of ES lines with specific shRNA constructs was shown by the expression of specific chondrogenic marker genes *COL10A1*, *IHH* and *SOX9* using qRT-PCR. Data are mean ± SEM; *t*-test. (**B**) Analysis of *RUNX2* expression in A673, EW7 and SK-N-MC cells after transient combined HOXD knock down with specific siRNAs against *HOXD10*, *HOXD11* and *HOXD13* using qRT-PCR. Data are mean ± SEM; *t*-test. Middle panel western-blot of RUNX2 expression after triple HOXD knock down compared to respective controls (si.control: c). (**C**) mRNA analysis of *BGLAP* and *PDGFB* expression in 3 different ES lines after inhibition of *HOXD10*, *HOXD11* or *HOXD13* expression with specific shRNA. Data are mean ± SEM; *t*-test. (**D**) Expression analysis of *PTHLH* in ES after knock down of individual *HOXD* genes by qRT-PCR. Data are mean ± SEM; *t*-test.

First, we investigated whether posterior *HOXD* genes may affect chondrogenic differentiation associated genes by incubating ES lines stably infected with *HOXD10*, *HOXD11* or *HOXD13* shRNA containing retroviruses (see Supplementary Material, Figure S3B) with specific differentiation media. The differentiation potential was analyzed using established marker genes, *COL10A1* (*collagen, type X, alpha-1*), *IHH* (*indian hedgehog*) and *SOX9* (*SRY-box 9*) for chondrogenic differentiation [34]. As shown in Figure 3A, early chondrogenic differentiation potential was impaired in SK-N-MC cells after HOXD10 and HOXD11 knock down as demonstrated by the decrease of IHH and SOX9 induction. In contrast, late chondrogenic differentiation as evaluated by COL10A1 seemed unaffected. Results for HOXD10 were reproducibly observed after knock down in A673 cells (see Supplementary Material, Figure S4A), while HOXD13 suppression seemed to support SOX9 expression during chondrogenic differentiation of SK-N-MC cells (Figure 3A), but these results were not reproducible in A673 cells (see Supplementary Material, Figure S4A). However, posterior *HOXD* genes seemed not to affect osteogenic differentiation, since no significant differences in Alizarin Red S staining (see Supplementary Material, Figure S4B) or osteogenic marker gene expression were observed (see Supplementary Material, Figure S4C).

Posterior *HOXD* genes are known to affect RUNX2 expression during ossification [22] and may do so via direct interaction of individual posterior *HOXD* genes [35]. In fact, when we analyzed individual ES lines after a triple knock down of HOXD10, HOXD11 and HOXD13 (Figure 3B, top) we reproducibly observed a clear inhibition of RUNX2 expression under normal culture conditions (Figure 3B, bottom). Interestingly, RUNX2 expression was not consistently affected after single knock down of each *HOXD* gene (data not shown) even after incubation in osteogenic differentiation media (see Supplementary Material, Figure S4C). But, posterior *HOXD* genes clearly promoted the expression of additional genes important for ossification or endochondral bone development in ES such as osteoblast-specific gene *osteocalcin* (*bone gamma-carboxyglutamate (gla) protein*, *BGLAP*), the pre-/osteoclast specific factor *platelet-derived growth factor beta polypeptide* (*PDGFB*) [36] (Figure 3C) or induction of osteolytic ES growth typical *PTHLH* [5] (Figure 3D), respectively.

### *HOXD* genes enhance TRAP^+^ osteoclasts in osteotropic tumor growth

**Figure 4:**
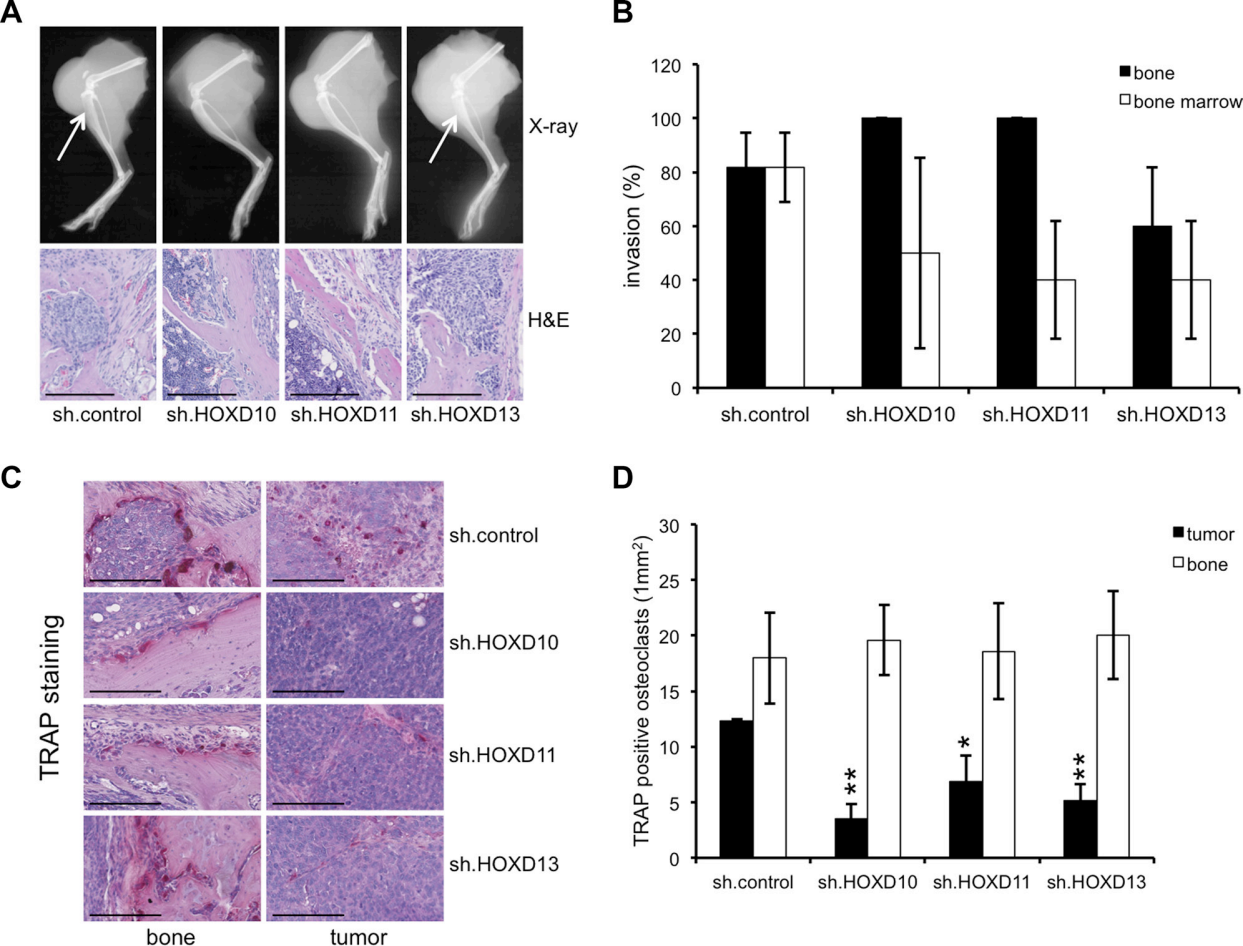
*HOXD* genes enhance the amount of TRAP^+^ osteoclasts in the tumor Analysis of bone invasiveness and osteolysis of constitutive A673 *HOXD10*, *HOXD11* and *HOXD13* shRNA infectants and negative controls in an orthotopic bone xeno-transplantation model (5–11 mice/group). Affected bones were assessed by X-ray radiography and histology. (**A**) Representative pictures of X-ray radiography and H&E staining showing no clear differences in bone invasiveness between A673 cells with constitutive HOXD10, HOXD11 or HOXD13 knock down and respective controls (X-ray, H&E, scale bar 0.25 mm). (**B**) Percentage of mice exhibiting infiltration of cortical bone or bone marrow infiltration after intra-tibial injection. (**C**) TRAP staining of osteoclasts for better visualization and to quantify osteolysis in bone and tumor tissues (scale bar 0.15 mm). (**D**) Average number of TRAP^+^ osteoclasts attached to the bone or in the tumor (1 mm^2^) was determined in at least three tumor samples/group (In each sample not less than 20 segments were analyzed).

DKK2 was previously identified to be a critical mediator of osteolytic tumor growth in ES [5]. Here, we asked whether the effect of DKK2 is mediated via the activity of posterior *HOXD* genes and if these genes may influence bone invasion and osteolysis *in vivo*, too. We injected A673 cells stable infected with *HOXD10*, *HOXD11* and *HOXD13* shRNA into the tibiae of immunodeficient Rag2^−/−^γ_C_^−/−^ mice and analyzed bone infiltration and destruction by X-ray radiography and histology. In contrast to DKK2, stable knock down of *HOXD* genes did not significantly influence bone invasiveness, although suppression of HOXD10, HOXD11 and HOXD13 seemed to slightly reduce the invasive growth potential of tumor cells in the bone marrow (Figure 4A and 4B). Especially after injection of constitutive A673 sh.HOXD10 and sh.HOXD11 transfectants, the amount of tumor cells in the bone marrow was clearly reduced (Figure 4A). Furthermore, we observed no considerable differences in osteolytic bone destruction after suppression of several posterior *HOXD* genes, as measured by the quantification of TRAP^+^-stained osteoclasts in the bone. Interestingly, HOXD10, HOXD11 and HOXD13 knock down significantly decreased the number of TRAP^+^ cells within tumor tissue while their number in bones was not affected (Figure 4C and 4D).

Subsequent expression analysis of key players associated with preparing the pre-metastatic niche, homing and invasion to bone as well as the osteolytic growth potential [37] demonstrated in addition to PTHLH (Figure 3D) only reduced gene expression of *interleukin 6* (*IL6*) after HOXD10 knock down (see Supplementary Material, Figure S5A). In contrast, *hypoxia-inducible factor 1, alpha subunit* (*HIF1*α) was reproducibly up-regulated after suppression of HOXD13 in different ES lines (see Supplementary Material, Figure S5A).

### *HOXD* genes promote ES growth and invasiveness

**Figure 5:**
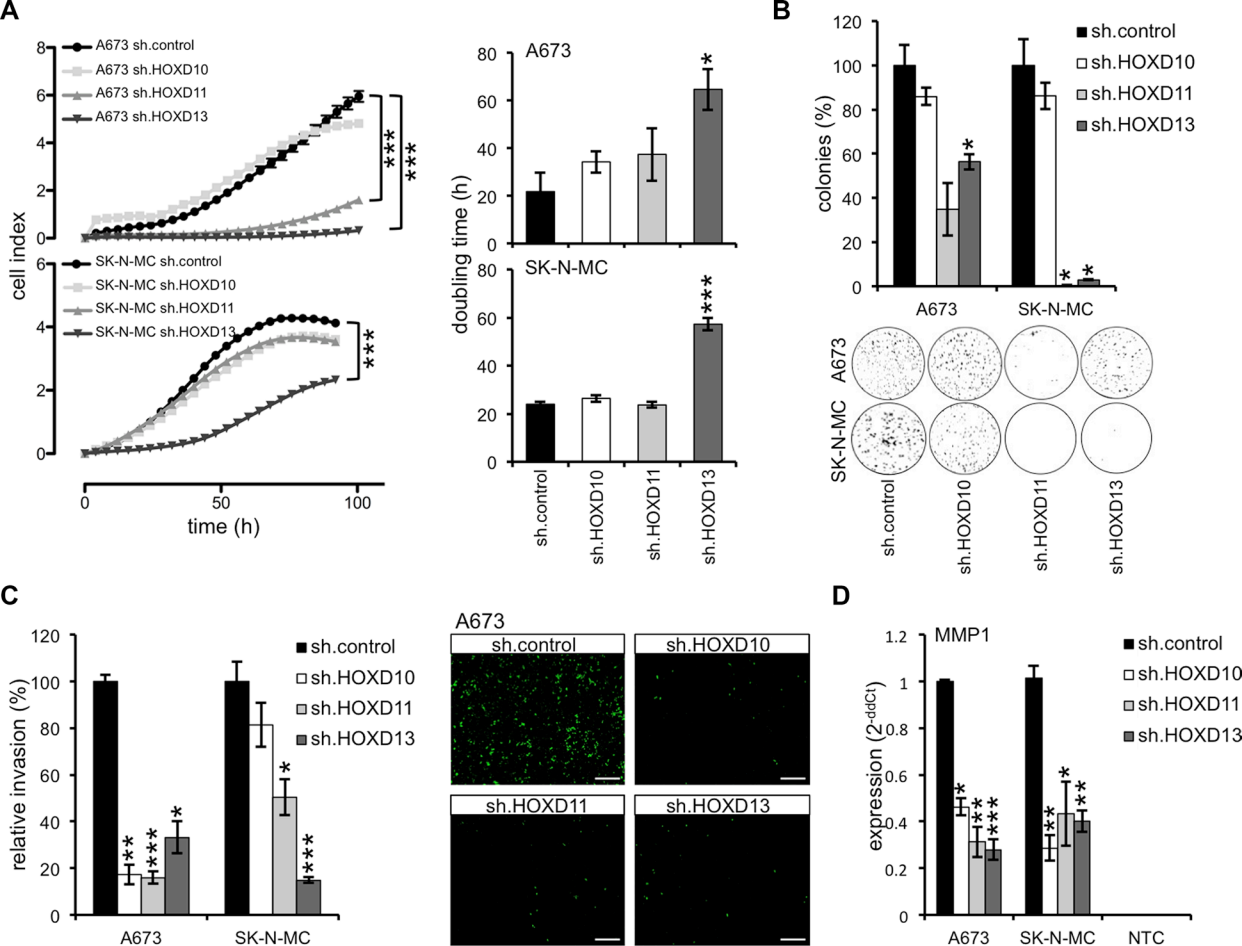
HOXD10, HOXD11 and HOXD13 promote growth and invasiveness of ES (**A**) Left, analysis of proliferation of constitutively infected ES lines with xCELLigence (hexaplicates/group). Cellular impedance was measured every 4 hours (relative cell index). Right, doubling time of constitutive A673 and SK-N-MC *HOXD10*, *HOXD11* and *HOXD13* shRNA infectants and respective controls (sh.control). Data are mean ± SEM of two independent experiments/cell line (hexaplicates/group). (**B**) Anchorage-independent colony formation in methylcellulose of ES lines with stable HOXD10, HOXD11 and HOXD13 knock down. Upper panel, data are mean ± SEM of two independent experiments (duplicates/group). Lower panel, macrographs show two representative experiments with A673 and SK-N-MC. (**C**) Analysis of invasiveness of ES lines through Matrigel after transfection with specific *HOXD10*, *HOXD11* or *HOXD13* shRNA constructs. Left, data are mean ± SEM of three independent experiments. Right, invasive A673 cells are shown after 48 hours incubation (scale bar 500 μm). (**D**) Expression of *MMP1* mRNA after HOXD knock down (sh.HOXD10, sh.HOXD11 and sh.HOXD13) using qRT-PCR. Data are mean ± SEM; *t*-test.

To further elucidate the possible contribution of posterior *HOXD* genes to phenotype and tumor pathology of ES we analyzed contact dependent growth of *HOXD10*, *HOXD11* and *HOXD13* shRNA infectants with an impedance-based system. As shown in Figure 5A, we observed a significant inhibition of proliferation after suppression of HOXD13 expression in A673 and SK-N-MC cells, whereas no significant, reproducible contribution of HOXD10 and HOXD11 in this assay was detected. In colony forming assay in methylcellulose a strong reduction of colony formation after HOXD13 knock down, but also a significant contribution of HOXD11 to contact independent growth was observed (Figure 5B).

To assess phenotypic markers associated with malignancy, we asked whether posterior *HOXD* genes may influence *in vitro* invasiveness, too. To do this, ES lines were analyzed *in vitro* on BioCoat invasion plates and cells that invaded into the Matrigel and migrated to the other side of the membrane were monitored 48 hours later. As shown in Figure 5C, A673 and SK-N-MC *HOXD10*, *HOXD11* and *HOXD13* shRNA infectants revealed a significant contribution of at least HOXD11 and HOXD13 to *in vitro* invasiveness. Recent results of our laboratory already indicated a strong contribution of MMP1 to invasion and metastasis of ES and its possible induction via several, presumably independent pathways [5, 38, 39]. Subsequent analysis of MMP1 expression in these *HOXD* shRNA infectants demonstrated a strong induction of MMP1 expression by all three *HOXD* genes (Figure 5D, see Supplementary Material, Figure S5B) signifying that the reduced invasive potential of HOXD11 or HOXD13 and probably HOXD10 silenced ES cells may be mediated at least in part via MMP1. In contrast to MMP1, mRNA expression of other matrix metallopeptidases (namely *MMP7* or *MMP9*) were not affected after constitutive knock down of different *HOXD* genes (see Supplementary Material, Figure S5C).

### HOXD11 and HOXD13 promote metastatic spread *in vivo*

**Figure 6:**
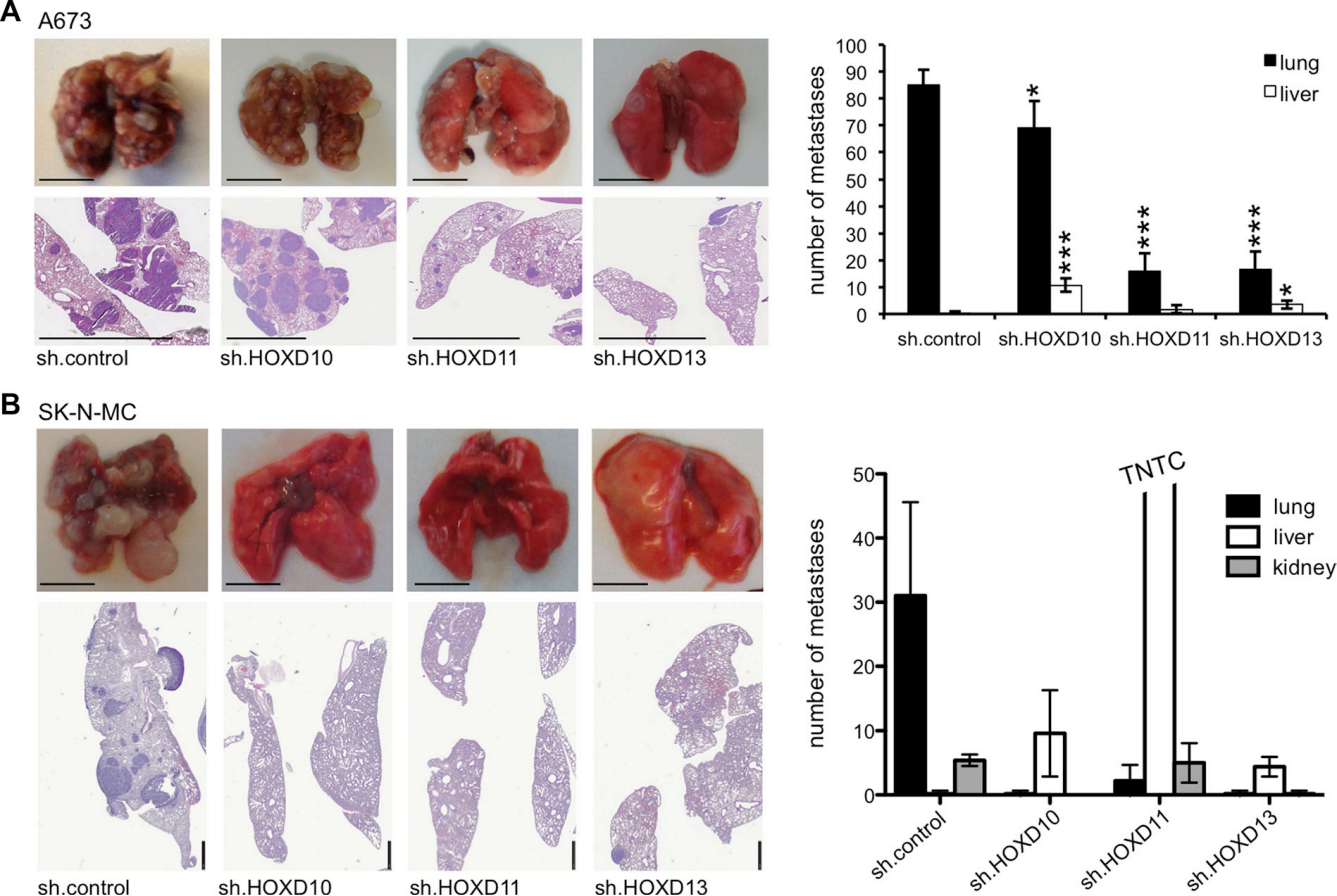
HOXD11 and HOXD13 promote lung metastasis in ES (**A**) *In vivo* analysis of the metastatic potential of A673 cells constitutively transfected with sh.control, sh.HOXD10, sh.HOXD11 and sh.HOXD13 in Rag2^−/−^γ_C_^−/−^ mice (5 mice/group). Affected organs were photographed and analyzed by histology. Left, representative pictures of whole organs and H&E staining sections are shown (scale bar 5 mm). Right, average number of apparent metastases in lung and liver tissues is plotted. (**B**) Similar experiments were carried out with SK-N-MC cells constitutively transfected with sh.control, sh.HOXD10, sh.HOXD11 and sh.HOXD13 in immune deficient Rag2^−/−^γ_C_^−/−^ mice (5 mice/group). Here knock down of HOXD10 also inhibited lung metastasis (scale bar 5 or 1mm).

Since HOXD11 and HOXD13 contributed to ES *in vitro* invasiveness we finally asked whether posterior *HOXD* genes may promote metastatic potential of ES *in vivo*. Thus, we injected stable *HOXD10*, *HOXD11* and *HOXD13* shRNA infected A673 cells and appropriate controls into the tail vein of Rag2^−/−^γ_C_^−/−^ mice. As shown in Figure 6A left, A673 control infectants grew to numerous confluent and necrotic tumor nodules within the lung, while A673 sh.HOXD11 and A673 sh.HOXD13 infectants revealed a significantly reduced metastatic phenotype that was statistically significant (Figure 6A, right). *HOXD10* shRNA infectants formed lung tumors indistinguishable from control infectants with increased appearance of tumor nodules within the liver (Figure 6A, right). After serial sectioning, we observed that A673 sh.HOXD10 and sh.control tumor nodules in lungs were bigger with a higher amount of necrosis than the tumor nodules after injection of A673 cells with stable HOXD11 and HOXD13 knock down. These results were confirmed with SK-N-MC cells (Figure 6B): shRNA-mediated knock down of HOXD10, HOXD11 or HOXD13, respectively, resulted in a strong inhibition of lung metastasis (Figure 6B), while there was some unspecific increase of liver metastasis in this mouse model. This overall indicates that especially HOXD11 and HOXD13 are important for lung metastatic potential in ES.

## DISCUSSION

Ewing sarcoma (ES) a bone and soft tissue malignancy is characterized by early metastasis to lung and bone [1]. Here we observed genes such as *HOXD10*, *HOXD11* and *HOXD13* normally involved in bone formation and ossification pattern of bones [22], to be significantly over-expressed in ES. Other genes of the posterior *HOXD* locus including *HOXD9* and *HOXD12* where not uniquely up-regulated in primary ES compared to other tumors.

In search for factors presumably involved in their increased expression in ES we investigated putative ES originating cells such as neural crest or mesenchymal stem cells [1–3]. Though, genes of the *HOXD* locus were already expressed in mesenchymal or neural crest derived cells (Figure 1B), they were not increased early after up-regulation of EWS-FLI1 in such stem cells nor did EWS-FLI1 knock down in ES lines influence HOXD10, HOXD11 or HOXD13 expression *in vitro*.

Remarkably, we identified HOXD10, HOXD11 and HOXD13 to be potential downstream targets of DKK2 using RNA interference analysis (Figure 1F). We previously demonstrated DKK2 to be an agonist of the canonical WNT/β-catenin pathway and critical mediator of osteolytic tumor growth in ES [5]. Here, analysis of mesenchymal and neuroectodermal stem cells demonstrated an up-regulation of DKK2 in EWS-FLI1 expressing neuroectodermal stem cells [27]. Long-term exposure in differentiation media resulted in a significant further up-regulation of DKK2 and HOXD13 in EWS-FLI1^+^ stem cells. DKK2 dependency of posterior *HOXD* gene expression persisted in ES lines and was further increased after transfection of DKK2 encoding cDNA into ES. In addition, treatment of ES lines with ligands of the canonical WNT/β-catenin signaling pathway resulted in an additional up-regulation of posterior *HOXD* genes as well as of the WNT/β-catenin target gene *LEF1.* This overall indicates a biologically active WNT signaling pathway in ES involved in posterior *HOXD* gene induction and expression.

Previous results already demonstrated agonists of WNT/β-catenin signaling to support skeletogenesis [40] or to play a role in terminal osteoblast differentiation into mineralized bone matrices [41]. HOXD11, HOXD12 and HOXD13 were shown to not only regulate patterning but also to directly influence bone formation and the ossification pattern of bones [22], with HOXD13 presumably being a master regulator of autopod skeletal morphogenesis [26]. In line with these results we observed early chondrogenic differentiation potential to be significantly impaired after HOXD10 and HOXD11 knock down as demonstrated by the decrease of IHH and SOX9 induction. Furthermore, posterior *HOXD* gene expression similarly stimulated RUNX2 expression, and enhanced the expression of osteoblast-specific osteocalcin (BGLAP), pre-/osteoclast specific PDGFB and PTHLH. The transcription factor RUNX2 binds to the oncogenic fusion protein EWS-FLI1 [42] and may promote osteogenic differentiation and development of perichondrial cells that differentiate directly into osteoblasts in diaphysal bones [43]. RUNX2 further affects cancer cell invasion and osteolysis [37, 44], which seems also true for ES as demonstrated by us in the context of DKK2 [5]. However, after suppression of posterior *HOXD* genes only a slight reduction of bone marrow invasiveness, but an obvious reduction of TRAP^+^ osteoclasts within tumor tissue was observed. This supports a critical role for posterior *HOXD* gene mediated regulation of RUNX2 and osteolytic capacity of ES itself, although the underlying, exact mechanisms are not understood [37]. PDGFB, although osteoclast-specific, has been shown to contribute to bone development [36]. Characteristic PTHLH expression was observed in osteochondrogenic progenitor cells highly susceptible for EWS-FLI1 mediated transformation [6]. These results overall suggest that posterior *HOXD* genes in ES mimic an immature endochondral developmental transcriptional program presumably important for bone associated tumor growth and metastasis.

The posterior *HOXD* gene-mediated expression program in ES not only affected the immature differential but similarly the invasive, metastatic phenotype of ES. Several *HOX* genes were previously implicated in neoplastic transformation resulting in leukemia [23] as well as solid cancers derived from various organs [24, 25]. Furthermore, the involvement of particular *HOX* genes such as *HOXC13*, *HOXD3*, *HOXA1* in metastasis and invasiveness was recently demonstrated for melanoma, breast and prostate cancer, respectively [45–47].

While in ES HOXD11 and especially HOXD13 promoted *in vitro* proliferation and contact independent growth, *in vitro* invasiveness of ES lines was dependent on HOXD10, HOXD11 as well as HOXD13. The enhanced invasive potential seemed mediated at least in part via MMP1, since down-regulation of individual *HOXD* genes likewise resulted in a repressed MMP1 expression profile. MMP1 is well described as an AP1 target gene [48] and additional results of our laboratory demonstrated a STAT1 dependency of MMP1 expression in ES [38]. Furthermore, there is also evidence that MMP1 is a direct target of *EWS*/*ETS* proteins, as a previous study has shown that EWS-ETV1 (ER81) and EWS-FLI1 fusion proteins can interact with the MMP1 promoter and collaborate with c-Jun and the cofactor p300 to activate *MMP1* gene transcription *in vitro* [49]: But in our hands, EWS-FLI1 knock down suppressed MMP1 expression only in A673 cells but not in other ES lines. Previous results of our laboratory already indicated a strong contribution of MMP1 to metastasis of ES and its possible induction via several, presumably independent pathways that so far was not understood [5, 38, 39]. In line with these observations, knock down of HOXD11 or HOXD13 significantly suppressed metastasis in a xeno-transplant model in immune deficient Rag2^−/−^γ_C_^−/−^ mice. So expression of posterior *HOXD* genes in ES may generate an open transcriptional platform for MMP1 induction, that may be activated by multiple pathways, sensing e.g. oxidative stress via STAT1 [38] or other factors of the microenvironment [39]. However, details of these pathway interplays have to be further investigated.

In summary, posterior *HOXD* genes over-expressed and presumably deregulated via DKK2 and the canonical WNT/β-catenin pathway in ES seem critical mediators of an immature endochondral program of ES mediating a transcriptional profile important for ES bone malignancy and its metastatic potential.

## MATERIALS AND METHODS

### Cell lines

Cells were maintained in a humidified incubator at 37°C in 5–8% CO_2_ atmosphere in RPMI 1640 or DMEM medium (both Invitrogen) containing 10% heat-inactivated fetal bovine serum (FBS, Biochrom) and 100μg/ml gentamicin (Invitrogen). Cell lines were checked routinely for purity (e.g. EWS-FLI1 translocation product, surface antigen or HLA-phenotype) and Mycoplasma contamination.

### Expression profiling

For comparative gene expression analysis of individual *HOXD* genes, RNA of 13 individual ES and 12 NB were hybridized onto HG U133A arrays (Affymetrix; GSE1825, GSE15757; [50]) and compared to a published microarray study of 36 normal tissues (NT) (GSE2361) of diverse origin including tissues of normal heart, thymus, spleen, ovary, kidney, skeletal muscle, pancreas, prostate, small intestine, colon, placenta, bladder, breast, uterus, thyroid, skin, salivary gland, trachea, cerebellum, brain, fetal brain, adrenal gland, bone marrow, amygdala, caudate nucleus, corpus, hippocampus, thalamus, pituitary gland, spinal cord, testis, liver, stomach, lung, fetal lung and fetal liver. All datasets were analyzed by using Microarray Suite 5.0, and scaled to the same target intensity of 500. Whole genome expression profiling of undifferentiated human embryonic stem cell (hESC)-derived neural crest cells, with and without EWS-FLI1, and adult bone marrow derived mesenchymal stem cells was performed as previously described [27], and data were downloaded from GEO (GSE 21511). To evaluate gene expression in neural crest stem cells that had been exposed to differentiation conditions, control and EWS-FLI1-transduced NC-MSC were transferred to serum containing differentiation media (Data are available at GEO GSE68898).

### RNA interference

For transient RNA interference cells were transfected with small interfering RNA (siRNA) as previously described [28]. siRNA sequences are provided in the supplementary data.

### Constructs and retroviral gene transfer

The production of stable infectants was done as described by Richter *et al.* [28].

### Quantitative RT-PCR

Differential gene expression was analyzed by qRT-PCR using TaqMan^TM^ Universal PCR Master Mix and fluorescence detection with an AB7300 Real-Time PCR System (both Life Technologies) as previously described [28]. A list of used assays is provided in the supplementary data. NTC: non template control.

### Constructs and transfection

Human cDNA ORF clone of DKK2 (NM_014421; OriGene) was transfected via electroporation into human cell lines. Stable transfectants were isolated after selection in 600μg/ml G418 (Sigma Aldrich).

### WNT ligand stimulation

To analyze a possible involvement of WNT signaling triggered by WNT ligands on *HOXD* expression, we incubated A673, SK-N-MC and TC-71 with two different concentrations of recombinant human WNT3a (5036-WN, 0.1μg/ml or 0.3μg/ml), WNT5a (645-WN, 0.1μg/ml or 0.3μg/ml) or WNT11 (6179-WN, 0.6μg/ml or 1.2mg/ml; all R&D Systems). Additionally, we analyzed each ligand in combination. After 1, 3, 6 and 12h RNA was isolated and mRNA expression levels of *HOXD10*, *HOXD11*, *HOXD13* and the WNT target gene *LEF1* were analyzed by qRT-PCR.

### Differentiation assay

For testing of chondrogenic cell differentiation, cells were cultured in specific differentiation media (STEMPRO Chondrogenesis Differentiation Kit, Invitrogen) according to the manufacturer's instructions. To validate differentiation efficacy, expression of the well-known chondrogenic marker genes *COL10A1, IHH* and *SOX9* was monitored by qRT-PCR [34].

### Proliferation assay

Cell proliferation was measured according to the manufacturer with an impedance-based instrument system (xCELLigence, Roche) enabling label-free real time cell analysis.

### Colony forming assay

Cells were seeded in duplicate into a 35mm plate at a density of 5×10^3^ cells per 1.5ml methylcellulose-based media (R&D Systems) according to the manufacturer's instruction and cultured for 14 days at 37°C/5% CO_2_ in a humidified atmosphere.

### *In vitro* invasion assay

To study cell invasion *BioCoat™ Angiogenesis System: Endothelial Cell invasion* was used (BD Biosciences) according to the manufacturer's instructions.

### Western blot

Procedures were described previously [28]. Following antibodies were used: anti-RUNX2 (mouse monoclonal, clone AS110, 05-1478 Millipore). Equal protein loading was controlled with rabbit polyclonal to HPRT antibodies (1:500; sc20975; Santa Cruz Biotechnology).

### Mice

Immune deficient Rag2^−/−^γ_C_^−/−^ mice on a BALB/c background were obtained from the Central Institute for Experimental Animals (Kawasaki, Japan) and maintained in our animal facility under pathogen-free conditions in accordance with the institutional guidelines and approval by local authorities. Experiments were performed in 6-16 week old mice.

### *In vivo* experiments

For the analysis of *in vivo* metastatic potential 2×10^6^ ES cells were injected in a volume of 0.2ml into the tail vein of immunodeficient Rag2^−/−^γ_C_^−/−^ mice. Five weeks later mice were sacrificed and metastatic spread was monitored in individual organs. To examine bone invasiveness and osteolysis, mice were anesthetized with 500mg/ml Novaminsulfon (Ratiopharm) and isoflurane (Abbott) and injection was done as previously described [5]. In all experiments, tumors and affected tissues were recovered and processed for histological analyses. Intra-tibial tumor formation was monitored by X-ray radiography.

### Histology

Visceral organs were fixed in phosphate buffered 4% formaldehyde and paraffin embedded. 3-5μm thick sections from all tissues were stained with hematoxylin and eosin (H&E). Hind limb bones were decalcified and paraffin embedded, the histological analysis with H&E was complemented by quantification of tartrate-resistant acid phosphatase (TRAP^+^) stained osteoclasts. All sections were reviewed and interpreted by two pathologists (J.C-W. and F.N.).

### Statistical analysis

Descriptive statistics is used to determine parameters like mean, standard deviation and standard error of the mean (SEM). Differences were analyzed by unpaired two-tailed student's *t*-test as indicated using Excel (Microsoft) or Prism 5 (GraphPad Software); *p* values < 0.05 were considered statistically significant (*p < 0.05; **p < 0.005; ***p < 0.0005).

## SUPPLEMENTARY FIGURES AND TABLES


