# Supplementary material for: The posterior HOXD locus: Its contribution to phenotype and malignancy of Ewing sarcoma
Source: Oncotarget. 2016 May 30;7(27):41767–80. doi: 10.18632/oncotarget.9702 (PMC5173095; doi:10.18632/oncotarget.9702)
Supplement: Supplementary file 1 [file oncotarget-07-41767-s001.pdf]

The posterior *HOXD* locus: Its contribution to phenotype and malignancy of Ewing sarcoma

Supplementary Materials

SUPPLEMENTARY MATERIALS AND METHODS

Cell lines

Osteosarcoma cell lines (HOS, MG-63, SaOS-2 and U2 OS) were kindly provided by Jan Smida and Michaela Nathrath, Institute of Pathology and Radiation Biology (Neuherberg, Germany) and ES line EW7 by Olivier Delattre, Institut Curie (Paris, France). A673 was purchased from ATCC (LGC Standards GmbH). The other ES lines (MHH-ES1, RD-ES, SK-ES1, SK-N-MC and TC-71) and neuroblastoma lines (SH-SY5Y and SIMA) were obtained from the German Collection of Microorganisms and Cell Cultures (DSMZ). Mesenchymal stem cells L87 and V54.2, kindly provided by Peter Nelson (Medizinische Klinik und Poliklinik IV, München, Germany), were immortalized with SV40 large T-antigen [1]. Retrovirus packaging cell line PT67 was from Takara Bio Europe/Clontech.

Small interfering RNAs used

siRNAs for EWS-FLI1 were synthesized at MWG Biotech and correspond to published sequences [2]. All other siRNAs were purchased from Qiagen. si.EWS-FLI1_I 5′-GCUACGGGCAGCAGAACCCUU-3′ (sense) and 5′-GGGUUCUGCUGCCCGUAGCU-3′ (antisense); si.EWS-FLI1_II 5′-GCAGAACCCUUCUUAUGACUU-3′ (sense) and 5′-GUCAUAAGAAGGGUUCUGCUUU-3′ (antisense); si.EZH2_val 5′-CCAUGUUUACAACUAUCAA-3′ (sense) and 5′-UUGAUAGUUGUAAACAUGG-3′ (antisense); si.EZH2_2 5′-GCAAAUUCUCGGUGUCAAA-3′ (sense) and 5′-UUUGACACCGAGAAUUUGC-3′ (antisense); si.HOXD10_1 5′-GGGUAACUAUUAUUGCGCAUG-3′ (sense) and 5′-CAUGCGCAAUAAUAGUUACCC-3′ (antisense); si.HOXD10_4 5′-CCUUCACCACCAACAUUAAUG-3′ (sense) and 5′-CAUUAAUGUUGGUGGUGAAGG-3′ (antisense); si.HOXD10_5 5′-GAACAGAUCUUGUCGAAUAUU-3′ (sense) and 5′-UAUUCGACAAGAUCUGUUCGG-3′ (antisense); si.HOXD11_3 5′-CGUCUGACUUCGCUAGCAAUU-3′ (sense) and 5′-UUGCUAGCGAAGUCAGACGGG-3′ (antisense); si.HOXD11_5 5′-CAACCUCACUGACCGGCAAUU-3′ (sense) and 5′-UUGCCGGUCAGUGAGGUUGAG-3′ (antisense); si.HOXD11_6 5′-GGCCGAGCGGAUCCUAAUAUU-3′ (sense) and 5′-AAUAUUAGGAUCCGCUCGGCC-3′ (antisense); si.HOXD13_1 5′-GAGAGUGCCUUACACCAAAUU-3′ (sense) and 5′-UUUGGUGUAAGGCACUCUCUU-3′ (antisense); si.HOXD13_2 5′-GAACCUAUCUGAGAGACAAUU-3′ (sense) and 5′-UUGUCUCUCAGAUAGGUUCGU-3′ (antisense); si.HOXD13_3 5′-CAGUAUAAAGGGACUUGAAGC-3′ (sense) and 5′-GCUUCAAGUCCCUUUAUACUG-3′ (antisense); si.TCF4_3 5′-CAGCUGUUUGGUCUAGAAATT-3′ (sense) and 5′-UUUCUAGACCAAACAGCUGTG-3′ (antisense); si.TCF4_5 5′-CGACAAGAAGGAUAUCAAATT-3′ (sense) and 5′-UUUGAUAUCCUUCUUGUCGTC-3′ (antisense) and si.control 5′-UUCUCCGAACGUGUCACGU-3′ (sense) and 5′-ACGUGACACGUUCGGAGAA-3′ (antisense).

Short hairpin RNA coding oligonucleotides

DKK2 (sh.DKK2) 5′-GATCCGGGGATTTGCTATCATAATATTCAAGAGATATTATGATAGCAAATCCCCTTTTTTCTAGAG-3′ (sense) and 5′-AATTCTCTAGAAAAAAGGGGATTTGCTATCATAATATCTCTTGAATATTATGATAGCAAATCCCCG-3′ (antisense); HOXD10 (sh.HOXD10) 5′-GATCCGGGGTAACTATTATTGCGCATTCAAGAGATGCGCAATAATAGTTACCCCTTTTTTCTAGAG-3′ (sense) and 5′-AATTCTCTAGAAAAAAGGGGTAACTATTATTGCGCATCTCTTGAATGCGCAATAATAGTTACCCCG-3′ (antisense); HOXD11 (sh.HOXD11) 5′-GATCCGCGTCTGACTTCGCTAGCAATTCAAGAGATTGCTAGCGAAGTCAGACGCTTTTTTCTAGAG-3′ (sense) and 5′-AATTCTCTAGAAAAAAGCGTCTGACTTCGCTAGCAATCTCTTGAATTGCTAGCGAAGTCAGACGCG-3′ (antisense); HOXD13 (sh.HOXD13) 5′-GATCCGGAACCTATCTGAGAGACAATTCAAGAGATTGTCTCTCAGATAGGTTCCTTTTTTCTAGAG-3′ (sense) and 5′-AATTCTCTAGAAAAAAGGAACCTATCTGAGAGACAATCTCTTGAATTGTCTCTCAGATAGGTTCCG-3′ (antisense); and control shRNA (sh.control) 5′-GATCCGTTCTCCGA-ACGTGTCACGTTTCAAGAGAACGTGACACGTTCGGAGAACTTTTTTCTAGAG-3′ (sense) and 5′-AATTCTCTAGAAAAAAGTTCTCCGAACGTGTCACGTTCTCTTGAAACGTGACACGTTCGGAGAACG-3′ (antisense).

Primers and assays used for qRT-PCR

For EWS-FLI1 detection, the following primers 5′-TAGTTACCCACCCCAAACTGGAT-3′ (sense), 5′-GGGCCGTTGCTCTGTATTCTTAC-3′ (antisense) and probe 5′-FAM-CAGCTACGGGCAGCAGAACCCTTCTT-TAMRA-3′ were designed. Inventoried TaqMan Gene Expression Assays (Life Technologies) were used for the genes *BGLAP* (Hs01587814_g1), *COL1A1* (Hs00164004_m1), *COL10A1* (Hs00166657_m1), *DKK2* (Hs00205294_m1), *EZH2* (Hs00544830_m1), *GAPDH* (Hs99999905_m1), *HIF1α* (Hs00153153_m1), *HOXD10* (Hs00157974_m1), *HOXD11* (Hs00360798_m1), *HOXD13* (Hs00968515_m1), *IFITM1* (Hs00705137_s1), *IHH* (Hs01081801_m1), *IL6* (Hs00985639_m1), *ISG15* (Hs00192713_m1), *LEF1* (Hs01547250_m1), *MMP1* (Hs00899658_m1), *MMP7* (Hs01042796_m1), *MMP9* (Hs00234579_m1), *PDGFB* (Hs00966522_m1), *PTHLH* (Hs00174969_m1), *RUNX2* (Hs00231692_m1), *SOX9* (Hs00165814_m1), *SP7* (Hs01866874_s1), *TCF4* (Hs00162613_m1), *WNT3A* (Hs00263977_m1), *WNT5A* (Hs00998537_m1), *WNT11* (Hs00182986_m1).

Differentiation assays

For testing of osteogenic cell differentiation, cells were cultured in specific differentiation media (STEMPRO Osteogenesis Differentiation Kit, GIBCO, Invitrogen) for three weeks at 37°C/5% CO_2 according to the manufacturer's instructions. To validate differentiation efficacy, the presence of calcific depositions was detected using Alizarin Red S staining and the expression of the well-known osteogenic marker genes, *collagen, type I, alpha-1 (COL1A1), runt-related transcription factor 2 (RUNX2*) and *Sp7 transcription-factor (SP7*; also known as *osterix*) was monitored by qRT-PCR [3].

Alizarin red S staining

After three weeks under differentiation media (STEMPRO Osteogenesis Differentiation Kit, GIBCO, Invitrogen), the cells in a 6-well plate were rinsed once with 1x Dulbecco's phosphate buffered saline and fixed in 4% formaldehyde solution for 30 minutes. Subsequently, cells were carefully washed twice with distilled water and incubated with 2% Alizarin Red S solution (pH 4.2) for 6 minutes. After staining, cells were washed five times with distilled water and photographed.

Microscopy

Stained cells were photographed with a Zeiss AxioCam MRm camera on a Zeiss Axiovert 100 microscope (Zeiss).

REFERENCES

1. Moosmann S, Hutter J, Moser C, Krombach F, Huss R. Milieu-adopted in vitro and in vivo differentiation of mesenchymal tissues derived from different adult human CD34-negative progenitor cell clones. Cells Tissues Organs. 2005; 179:91–101.
2. Dohjima T, Lee NS, Li H, Ohno T and Rossi JJ. Small interfering RNAs expressed from a Pol III promoter suppress the EWS/Fli-1 transcript in an Ewing sarcoma cell line. Mol Ther. 2003; 7:811–816.
3. Vater C, Kasten P, Stiehler M. Culture media for the differentiation of mesenchymal stromal cells. Acta biomaterialia. 2011; 7:463–477.

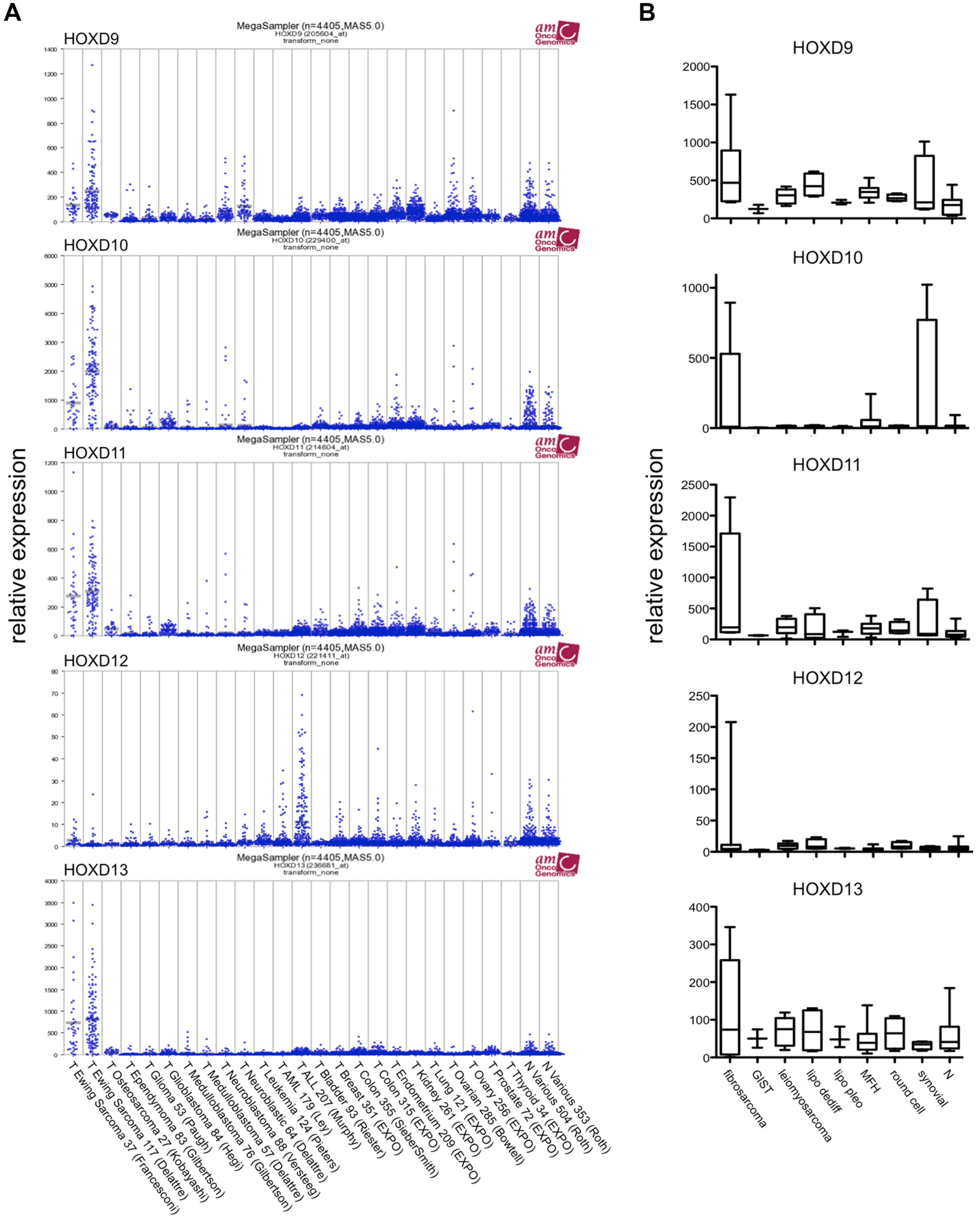

Supplementary Figure S1: Posterior *HOXD* gene expression in publically available expression data. (**A**) Expression levels of different *HOXD* genes in different pediatric small-round-blue-cell tumors, carcinomas and normal tissues by dot plot presentation using a comparative study of the amc onco-genomics software tool (www.amc.com). The number of samples in each cohort is given. (**B**) Gene expression profiles of posterior *HOXD* genes on 39 human sarcoma samples (GSM52571-GSM52609) and 15 control samples (GSM52556-GSM52570) were assessed using Affymetrix HG U133A oligonucleotide arrays (GSE2719).

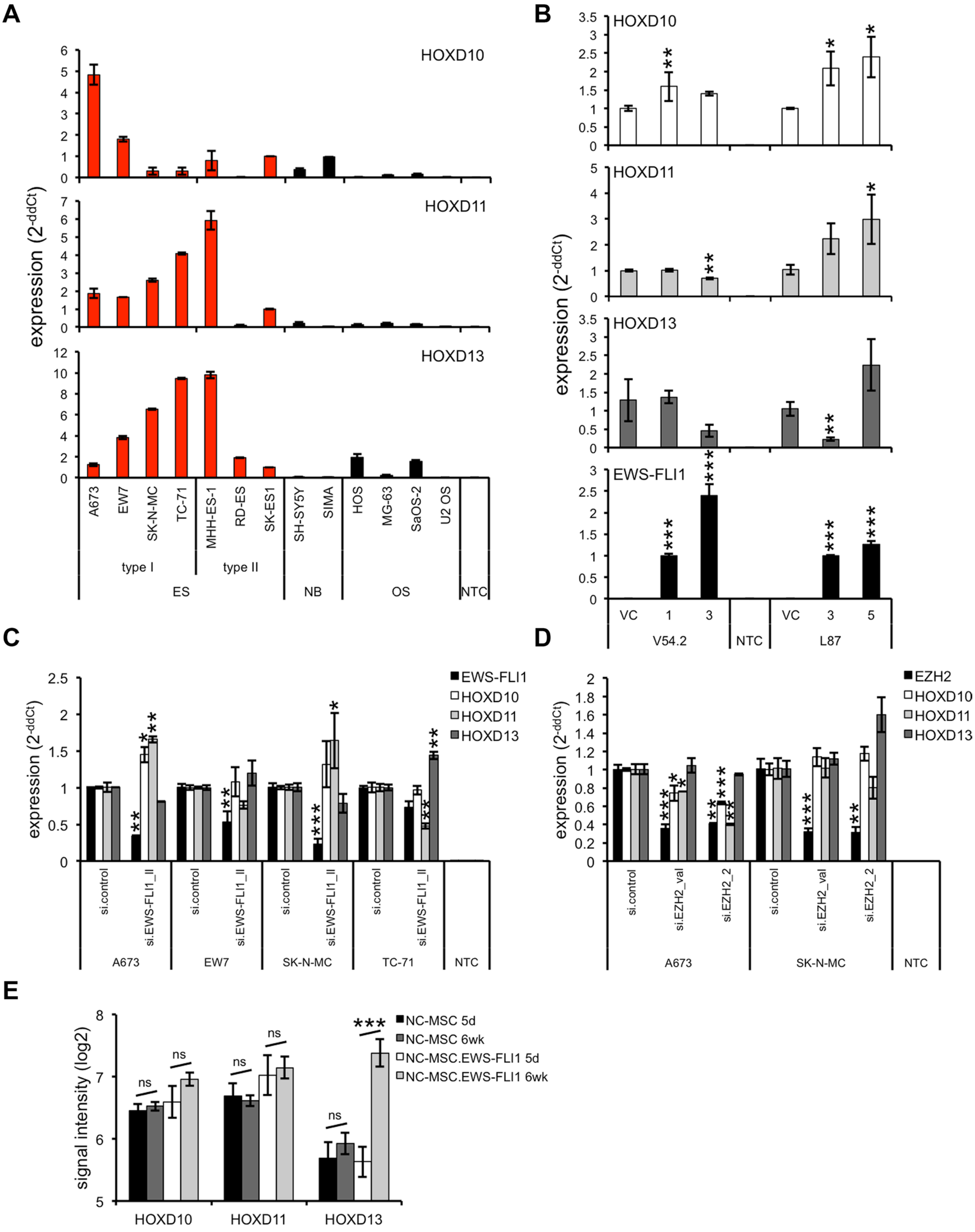

Supplementary Figure S2: *HOXD* gene expression. (**A**) *HOXD* gene expression in different ES, NB and osteosarcoma (OS) lines analyzed by qRT-PCR. Data are mean ± SEM; *t*-test. (**B**) Analysis of *EWS-FLI1*, *HOXD10*, *HOXD11* and *HOXD13* mRNA expression levels in mesenchymal stem cell lines V54.2 and L87 after transfection with either EWS-FLI1 expression vectors (numbers) or control vector (VC). Data are mean ± SEM; *t*-test. (**C**) qRT-PCR to detect *HOXD10*, *HOXD11* and *HOXD13* expression in four different ES cell lines after transfection with si.EWS-FLI1_II and si.control. Data are mean ± SEM; *t*-test. (**D**) EZH2 does not influence the expression of *HOXD10*, *HOXD11* or *HOXD13* in ES analyzed by qRT-PCR. Data are mean ± SEM; *t*-test. (**E**) Expression of HOXD10, HOXD11 and HOXD13 in NC-MSC cells following exposure to differentiation conditions. NC-MSC (neural crest-derived MSC) transduced with GFP-only (NC-MSC) or EWS-FLI1-GFP (NC-MSC.EWS-FLI1) lentiviral vectors were passaged for 5 days in self-renewal media (5 d) and then transferred to differentiation media for 6 weeks (6 wk). Gene expression profiling studies of triplicate samples reveals that exposure to differentiation conditions resulted in up-regulation of HOXD13 in EWS-FLI1^{+} cells.

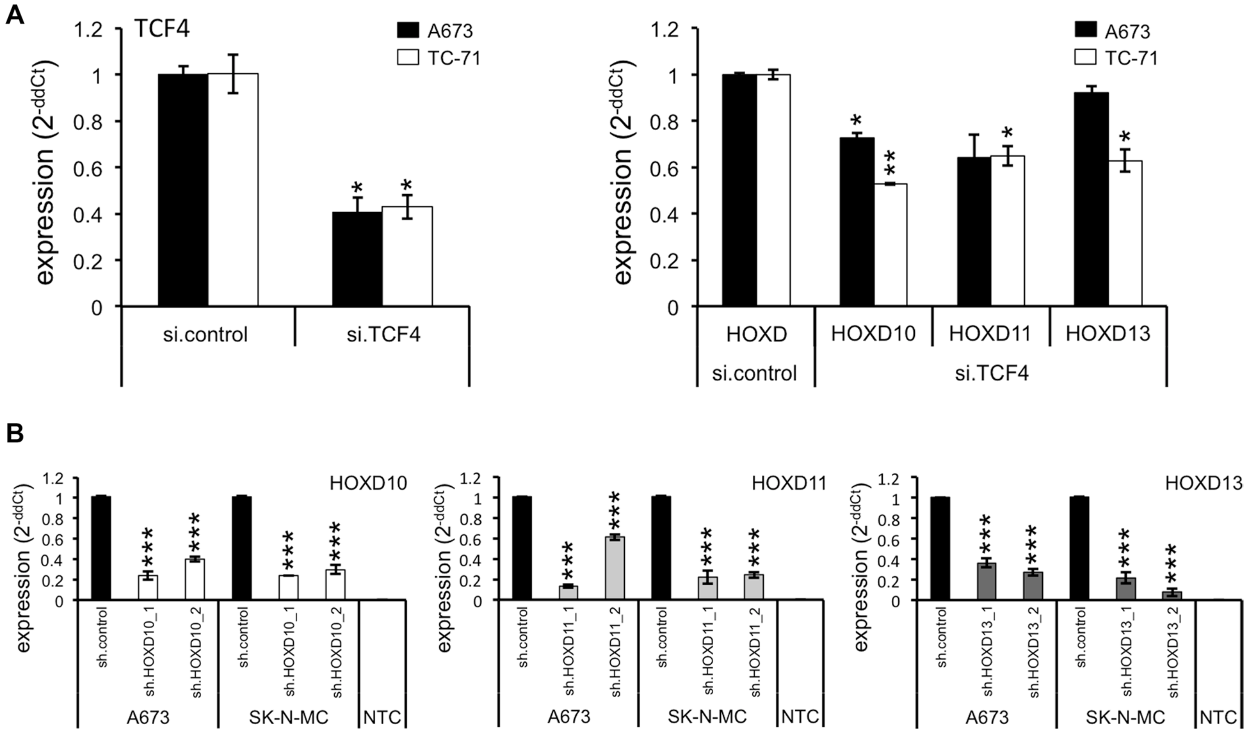

Supplementary Figure S3: Regulation of *HOXD* gene expression. (**A**) Down regulation of *HOXD10*, *HOXD11* or *HOXD13* expression after siRNA mediated knock down of TCF4. The results of combined qRT-PCR analyses are shown. Data are mean ± SEM; *t*-test. (**B**) Constitutive suppression of *HOXD10*, *HOXD11* or *HOXD13* expression after infection of ES cells with *HOXD* specific shRNA constructs as measured by qRT-PCR (sh.HOXD10, sh.HOXD11 or sh.HOXD13 and sh.control). Data are mean ± SEM; *t*-test.

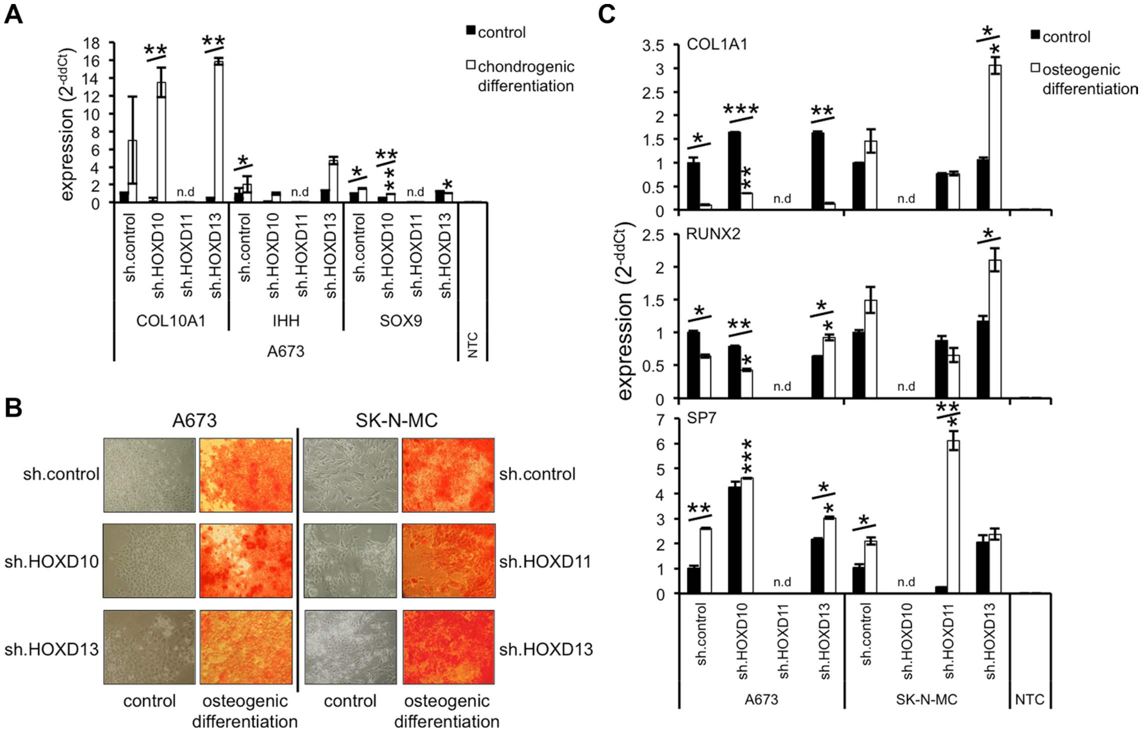

Supplementary Figure S4: Chondrogenic and osteogenic differentiation potential of ES cells after HOXD knock down. (**A**) Analysis of chondrogenic differentiation potential of A673 cells after stable HOXD knock down was shown by the expression of specific chondrogenic marker genes *COL10A1*, *IHH* and *SOX9* using qRT-PCR. Data are mean ± SEM; *t*-test. (**B**) Alizarin Red S staining of different ES lines with stable sh.HOXD10, sh.HOXD11, sh.HOXD13 and sh.control infectants cultivated for three weeks in osteogenic differentiation media or control media to detect the presence of calcific depositions (magnification 10×). (**C**) Osteogenic differentiation potential of ES lines with specific *HOXD* shRNA constructs was shown by the expression of specific osteogenic marker genes *COL1A1, RUNX2* and *SP7* using qRT-PCR. Data are mean ± SEM; *t*-test.

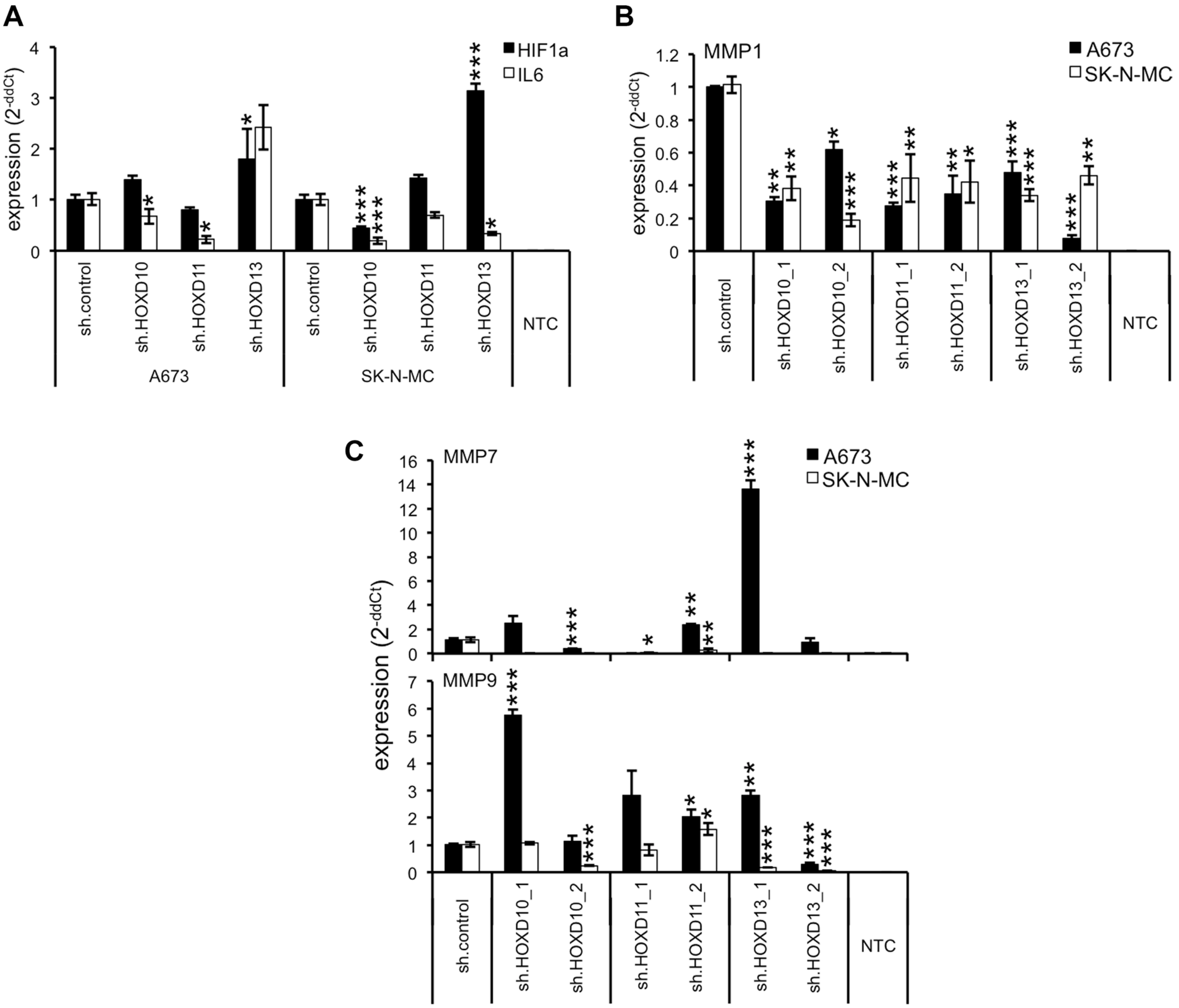

Supplementary Figure S5: Metastatic niche and MMP expression after stable HOXD knock down. (**A**) Gene expression analysis of two different osteolytic genes (*HIF1α* and *IL6*) in A673 and SK-N-MC cells constitutively transfected with *HOXD* shRNA (sh. HOXD10, sh.HOXD11, sh.HOXD13) and control shRNA (sh.control). Data are mean ± SEM; *t*-test. (**B**) *MMP1* expression in A673 and SK-N-MC cells stably transfected with sh.HOXD10, sh.HOXD11, sh.HOXD13 or sh.control shRNAs analyzed by qRT-PCR. Data are mean ± SEM; *t*-test. (**C**) Analysis of *MMP7* and *MMP9* mRNA levels after constitutive HOXD10, HOXD11 and HOXD13 knock down and respective controls. Data are mean ± SEM; *t*-test.

Supplementary Table S1: The 50 most up-regulated genes in ES compared to normal tissue

Probe set ID	Gene symbol	Gene description	FC	*p* value
**206645_s_at**	**NR0B1**	nuclear receptor subfamily 0, group B, member 1	61.87	0.0087
**207373_at**	**HOXD10**	homeobox D10	41.91	0.0026
**221854_at**	**PKP1**	plakophilin 1 (ectodermal dysplasia/skin fragility syndrome)	38.93	0.0070
**207397_s_at**	**HOXD13**	homeobox D13	28.42	0.0046
**206915_at**	**NKX2-2**	NK2 homeobox 2	26.89	0.0025
**61734_at**	**RCN3**	reticulocalbin 3, EF-hand calcium binding domain	26.75	0.0050
**206002_at**	**GPR64**	G protein-coupled receptor 64	26.16	0.0054
**219908_at**	**DKK2**	dickkopf WNT signaling pathway inhibitor 2	24.60	0.0063
**219825_at**	**CYP26B1**	cytochrome P450, family 26, subfamily B, polypeptide 1	18.34	0.0048
**219360_s_at**	**TRPM4**	transient receptor potential cation channel, subfamily M, member 4	17.95	0.0038
**218831_s_at**	**FCGRT**	Fc fragment of IgG, receptor, transporter, alpha	17.58	0.0004
**217303_s_at**	**ADRB3**	adrenoceptor beta 3	17.51	0.0017
**205131_x_at**	**CLEC11A**	C-type lectin domain family 11, member A	16.90	0.0027
**208712_at**	**CCND1**	cyclin D1	16.66	0.0002
**201291_s_at**	**TOP2A**	topoisomerase (DNA) II alpha 170kDa	16.35	0.0004
**210783_x_at**	**CLEC11A**	C-type lectin domain family 11, member A	16.26	0.0037
**215695_s_at**	**GYG2**	glycogenin 2	15.63	0.0022
**206812_at**	**ADRB3**	adrenoceptor beta 3	15.59	0.0015
**206025_s_at**	**TNFAIP6**	tumor necrosis factor, alpha-induced protein 6	15.19	0.0080
**208060_at**	**PAX7**	paired box 7	15.02	0.0022
**204669_s_at**	**RNF24**	ring finger protein 24	14.43	0.0004
**217818_s_at**	**ARPC4**	actin related protein 2/3 complex, subunit 4, 20kDa	14.34	0.0084
**206114_at**	**EPHA4**	EPH receptor A4	14.32	0.0028
**202747_s_at**	**ITM2A**	integral membrane protein 2A	13.41	0.0005
**210410_s_at**	**MSH5 /// MSH5-SAPCD1 /// SAPCD1**	mutS homolog 5 (E. coli) /// MSH5-SAPCD1 readthrough (NMD candidate) /// suppressor APC domain containing 1	13.39	0.0005
**209196_at**	**WDR46**	WD repeat domain 46	13.14	0.0000
**204766_s_at**	**NUDT1**	nudix (nucleoside diphosphate linked moiety X)-type motif 1	12.07	0.0000
**203558_at**	**CUL7**	cullin 7	12.04	0.0000
**212396_s_at**	**EMC1**	ER membrane protein complex subunit 1	11.76	0.0000
**219686_at**	**STK32B**	serine/threonine kinase 32B	11.47	0.0026
**214604_at**	**HOXD11**	homeobox D11	11.22	0.0002
**218445_at**	**H2AFY2**	H2A histone family, member Y2	11.20	0.0005
**220085_at**	**HELLS**	helicase, lymphoid-specific	11.19	0.0005
**213718_at**	**RBM4**	RNA binding motif protein 4	11.15	0.0001
**215043_s_at**	**GUSBP3 /// GUSBP9 /// SMA4 /// SMA5**	glucuronidase, beta pseudogene 3 /// glucuronidase, beta pseudogene 9 /// glucuronidase, beta pseudogene /// glucuronidase, beta pseudogene	10.87	0.0008
**206866_at**	**CDH4**	cadherin 4, type 1, R-cadherin (retinal)	10.29	0.0077
**220184_at**	**NANOG**	Nanog homeobox	10.23	0.0028
**205542_at**	**STEAP1**	six transmembrane epithelial antigen of the prostate 1	10.17	0.0020
**219976_at**	**HOOK1**	hook homolog 1 (Drosophila)	10.15	0.0009
**221270_s_at**	**QTRT1**	queuine tRNA-ribosyltransferase 1	10.14	0.0000
**219408_at**	**PRMT7**	protein arginine methyltransferase 7	9.85	0.0007
**219528_s_at**	**BCL11B**	B-cell CLL/lymphoma 11B (zinc finger protein)	9.78	0.0007
**203358_s_at**	**EZH2**	enhancer of zeste homolog 2 (Drosophila)	9.78	0.0009

**213089_at**	**LOC100272216**	uncharacterized LOC100272216	9.65	0.0017
**214692_s_at**	**JRK**	jerky homolog (mouse)	9.55	0.0005
**206487_at**	**SUN1**	Sad1 and UNC84 domain containing 1	9.16	0.0006
**213552_at**	**GLCE**	glucuronic acid epimerase	8.86	0.0009
**220233_at**	**FBXO17**	F-box protein 17	8.82	0.0049
**204545_at**	**PEX6**	peroxisomal biogenesis factor 6	8.78	0.0061
**208711_s_at**	**CCND1**	cyclin D1	8.57	0.0017

List of the 50 most up-regulated genes with the strongest over-expression in ES compared to normal tissue (GSE1825, GSE15757 and GSE2361). *HOXD* genes are highlighted in red. FC = fold change.
